# Developing functional hydrogels for treatment of oral diseases

**DOI:** 10.1002/SMMD.20240020

**Published:** 2024-07-25

**Authors:** Chuanhui Song, Rui Liu, Yile Fang, Hongcheng Gu, Yu Wang

**Affiliations:** ^1^ Department of Rheumatology and Immunology Institute of Translational Medicine Nanjing Drum Tower Hospital Affiliated Hospital of Medical School Nanjing University Nanjing China; ^2^ State Key Laboratory of Digital Medical Engineering School of Biological Science and Medical Engineering Southeast University Nanjing China

**Keywords:** hydrogel, oral disease, periodontitis, tissue engineering, tooth

## Abstract

Oral disease is a severe healthcare challenge that diminishes people's quality of life. Functional hydrogels with suitable biodegradability, biocompatibility, and tunable mechanical properties have attracted remarkable interest and have been developed for treating oral diseases. In this review, we present up‐to‐date research on hydrogels for the management of dental caries, endodontics, periapical periodontitis, and periodontitis, depending on the progression of dental diseases. The strategies of hydrogels for treating oral mucosal diseases and salivary gland diseases are then classified. After that, we focus on the application of hydrogels related to tumor therapy and tissue defects. Finally, the review prospects the restrictions and the perspectives on the utilization of hydrogels in oral disease treatment. We believe this review will promote the advancement of more amicable, functional and personalized approaches for oral diseases.

## Graphical abstract

Developing functional hydrogels for the treatment of oral diseases. The hydrogel‐based strategy for synergistic therapy involving dental caries, endodontics, apical periodontitis, periodontitis, oral mucosal diseases, salivary gland disease, and oral tumor.

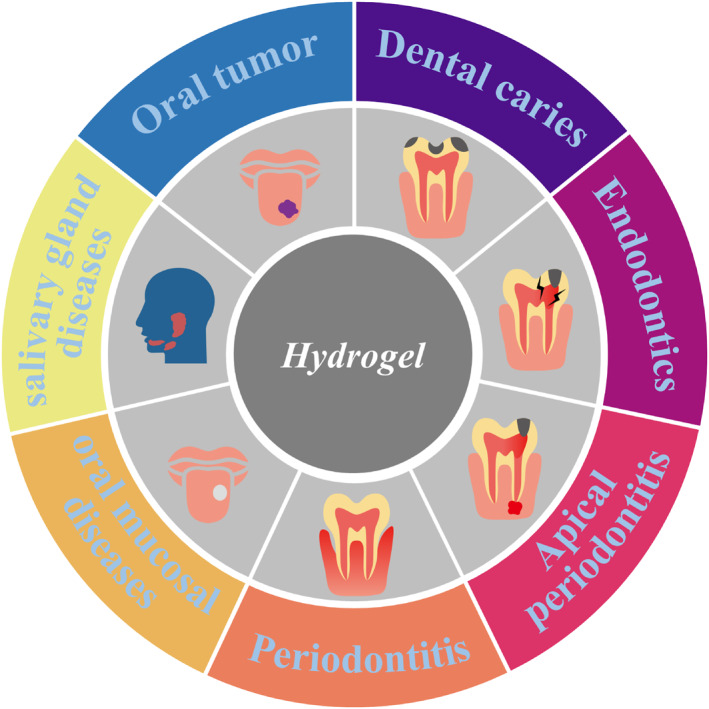

**Key points**

- Functional hydrogels involving the treatment of different oral diseases are introduced.
- The functionality of hydrogels and their typical medical applications for different disease characteristics are discussed.
- Current trends and future research directions of hydrogels in the treatment of oral diseases are prospected.

## 1 INTRODUCTION

As the start of the digestive tract, the oral cavity plays a huge role in daily life, and is responsible for mastication and speaking.^1^, ^2^ Due to its susceptibility to various diseases, ranging from infection to tumors, a great threat has been brought to human health and vitality.^3^, ^4^, ^5^, ^6^, ^7^ Traditional invasive operation is a commonly used clinical approach for addressing oral diseases. However, its application is limited, resulting from the sophisticated surgical skill requirements and inevitable patient pain.^8^, ^9^ Thus, a new strategy for comfortable oral therapy is urgently anticipated. With the development of materials and technologies, many biomedical materials owing to their multi‐functions, such as antibacterial, anti‐tumor, and osteogenic effects, have been developed, bringing brightness to oral disease treatment.^10^, ^11^ Among them, hydrogels show significant promise because of their good biocompatibility, predictable biodegradation rates, modifiable mechanical properties, and good flexibility.^12^, ^13^, ^14^, ^15^, ^16^, ^17^, ^18^, ^19^, ^20^ Attributed to this, they are regarded as promising carriers to combine with conventional medicine or newly developed novel drugs, which have demonstrated effective therapeutic effects to various oral diseases. Meanwhile, these oral diseases also put forward many requirements for hydrogels.

**FIGURE 1:**
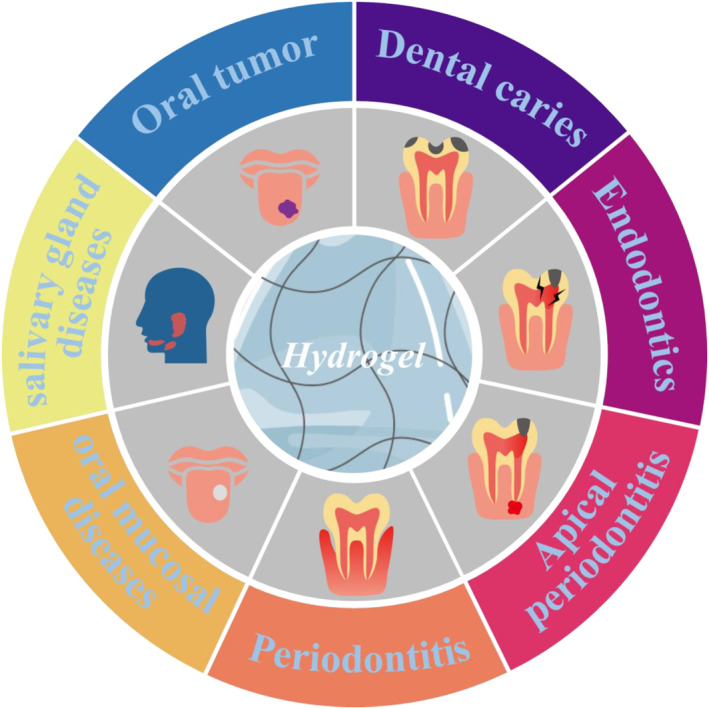
The hydrogel‐based strategy for synergistic therapy involves dental caries, endodontics, apical periodontitis, periodontitis, oral mucosal diseases, salivary gland disease, and oral tumors.

In this paper, we summarize the use of hydrogels in treating oral disease (Figure 1). According to the onset and progression of oral disease, we start with the hydrogels used to treat dental caries, endodontics, periapical periodontitis, and periodontitis. Then, we categorize the hydrogel‐involved strategy in treating oral mucosal and salivary gland diseases. Next, we review the relative application in the tumor treatment and tissue defect attempt to broaden the application fields of hydrogels. Finally, we take stock of the field and look ahead to current challenges and future directions.

## 2 HYDROGELS APPLICATION IN THE DENTAL CARIES

**FIGURE 2:**
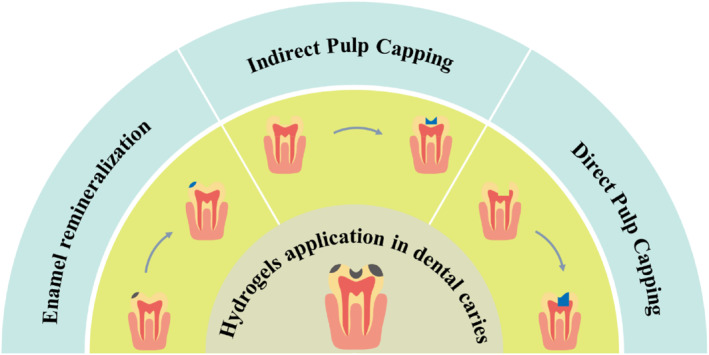
The application of hydrogels in the treatment of different stages of dental caries in different ways.

Dental caries (tooth decay) is a bacterial infection that provokes localized destruction of the hard tissues of the teeth and is one of the most common oral diseases worldwide.^21^ Acid produced by bacterial fermentation of carbohydrates in food is a direct cause of enamel demineralization, with *Streptococcusmutans (Staphylococcus mutans)* being the main cariogenic bacterium with robust acid production. Given this, one of the keys to dealing with dental caries is to control bacteria. Besides, the other vital aspect of caries prevention is to inhibit demineralization and promote remineralization. Fluoride has been widely proven to have anti‐caries effects, while its potential for fluorosis and staining limit its application. To overcome this issue, many agents like amelogenin‐derived peptides,^22^, ^23^ dentin phosphoprotein‐derived peptides,^24^ peptides with beta‐sheet structures^25^ and self‐assembling peptide (SAP)‐amphiphiles^26^ have been proposed, which can trigger biomimetic mineralization. To achieve effective therapeutic effects, hydrogels have been used as carriers to combine with various antibacterial and remineralization drugs or agents for the treatment of dental caries (Figure 2).

### 2.1 Antibacterial strategies

Many traditional antibacterial agents like silver have been applied to dental practice for centuries. Generally, silver amalgam materials are widely used for dental filling materials,^27^ in which silver shows a strong antibiotic effect against bacteria, both Gram‐negative and Gram‐positive,^28^ and can continuously prevent secondary caries. Due to stimulation of exopolysaccharides (EPS) synthesized by the interactions between *S*. *mutans* and glucosyltransferases, the biofilm is filmed and provides a stabilized sanctuary for the bacteria. Prolonging contact time with the lesion site will increase antimicrobial opportunities. Controlled drug release was demonstrated as a promising tool to achieve the goal. To realize controlled release, many novel biomaterials have been adopted and acted as silver carriers, such as poly vinyl alcohol (PVA),^29^, ^30^ alginate,^31^ and carboxymethyl cellulose (CMC),^32^ which can form hydrogels in aqueous settings. In addition, silver can be crosslinked with the nanocrystalline cellulose (CNC), showing controlled release capability, which extends antibacterial effects in dental practice.^33^ However, its potential poison and unfavorable coloring properties hinder its further application. Fortunately, there are many promising candidates for enhancing the antibacterial effect in dental settings.

In addition to strategies related to inorganic metal ions, organically based antimicrobial molecules have also made great progress recently. Among them, antimicrobial peptides (AMPs) have attracted much interest due to their excellent bactericidal properties and broad‐spectrum activity. Nowadays, AMPs are used in dental implants‐coatings^34^ and adhesive agents doping^35^, ^36^ to fight pathogens.^37^ Furthermore, considering the microbiota environment of the oral cavity, manipulating the oral microbiota is another useful antibacterial strategy. *Lactobacillus* is considered as the ideal candidate bacteria as they are active toward pathogens in the gastrointestinal tract and the oral cavity. In particular, *L*. *paracasei* 28.4 shows inhibition on *S*. *mutans*. To obtain the easy storage and long term live, the gellan hydrogel was taken as a vehicle to deliver living *L*. *paracasei* 28.4 to conquer *S*. *mutans*.^38^

### 2.2 Remineralization strategies

A popular strategy when caries is present is to perform a filling of the cavity after removal of the decayed tissue. However, the filling material is a polymer material that lacks biological activity and may lead to secondary caries. In recent decades, a strategy involving remineralization of the superficial dental tissue has received increasing attention as a noninvasive therapeutic technique, demonstrating its recognized therapeutic importance. Due to the designable functionality like great adhesive property, it is possible to rationally combine hydrogels with mineral remineralization compositions.

Dental enamel is a highly mineralized hard tissue composed of a highly ordered arrangement of hydroxyapatite (HA) nanocrystals. The most frequently used methods of remineralization include supplementation of mineral ions (i.e., calcium, phosphate, and fluoride) from dental varnish; filling of composites, glass ionomers, toothpastes, powders, or mouthwashes; and deposition of fluorapatite (FAP)‐like substances in enamel.^39^ The continuous contact time and flexible application site can obtain better mineralization results, which can be achieved by the hydrogel containing calcium, phosphate, and fluoride. The in vitro results showed that the agarose gel containing 0.26 M Na_2_HPO_4_ and CaCl_2_ can deposit HA crystals that are densely packed to each other, offering a simple method for achieving dentin remineralization.^40^ Further research employed hydroxypropylmethylcellulose (HPMC) as the remineralization composite carrier. After 24 h, this bio‐inspired mineralization film evoked early mineralization of the demineralized dentin, leading to increasing mineralization of the entire demineralized dentin (3–4 μm) after 72–96 h.^41^ As the most abundant enamel extracellular matrix protein, amelogenin is thought to play a crucial role in the formulation of hierarchically organized enamel crystals and amelogenin‐derived peptides. The research used agar as a hydrogel, which was mixed with amelogenin at a precise molar ratio. After remineralization with hydrogel, the microhardness of enamel surface was markedly increased. Notably, the full length amelogenin exerted an inhibitory effect on biofilms consisting of *S*. *mutans* and *Staphylococcus hemophilus*, but the underlined mechanism is unclear. Besides, the amelogenin showed excellent biocompatibility through culturing with gingival fibroblasts.^42^ It was reported that amelogenin could also be combined with chitosan (CS) hydrogel, which promoted rapid mineral induction and orderly growth of HA crystals.^43^

In addition to full‐length amyloid proteins, derived peptides such as QP5 can also be used as remineralizing elements in buffers.^44^ To mimic the natural extracellular matrix, integrating the remineralization element with the injectable hydrogels shows a remarkable ability for cavity preparation due to their shape programmability. The injectable hydrogel with maleic CS, thiolated alginate premixed with calcium b‐glycerophosphate and calcium carbonate exhibit outstanding highly crystalline Hap mineralization properties.^45^ Generally, it is challenging to accurately replicate the complicated hierarchical organization of high‐performance biomaterials in scalable biocomposites. By assembling amorphous intergranular phase (AIP)‐coated HA nanowires interwoven with PVA, it is possible to realize enamel analogs with a multiscale fundamentally hierarchical structure. The obtained nanocomposites exhibit simultaneous high rigidity, stiffness, intensity, viscoelasticity, and toughness, which outperform enamel and previously produce bulk enamel‐inspired materials.^46^ The hydrogel with the presence of CS may inhibit the bacteria because their protonated positive charge interacts with the bacterial cell wall, leading to bacterial aggregation and death, which directly or indirectly reduces biofilm biomass, lactate production, and metabolic activity.

## 3 HYDROGELS APPLICATION IN THE ENDODONTICS

The untreated‐dental caries may induce irreversible pulpitis, which results in the loss of pulpal tissue functions and entails root canal treatment. In addition, due to the active nature of pulpal tissue, the trauma‐induced endodontics has frequent symptoms, which poses a challenge for preserving the pulpal tissue. Thus, the infection ablation and regeneration are two critical methods in the treatment of endodontics. Owing to the limited space in the root canals, it is of great significance to be injectable or flowable. Compared with the traditional antibacterial preparation like paste and lotion, the hydrogel has unique advantages of injectability and adhesion, which can achieve controllable release and long‐time retention. Besides, the scaffold provided by the hydrogel can give the space for cell delivery.

**FIGURE 3:**
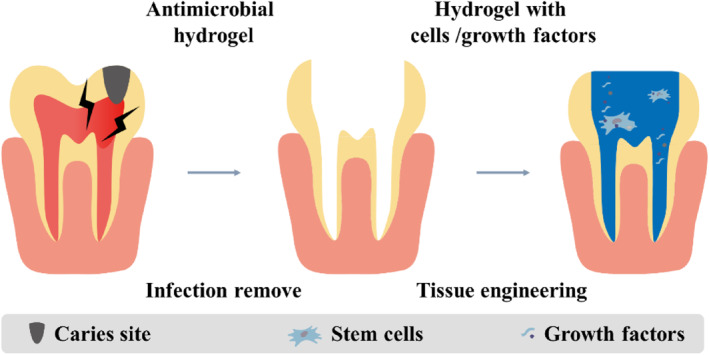
Hydrogel application in endodontics. The hydrogel can be applied in the process of infection removal and filling during the treatment of the endodontics.

There are two main categories of hydrogel‐based antimicrobial strategies: delivery of bactericidal substances and antimicrobial properties of the hydrogel components themselves. With the biocompatible and biodegradable photopolymerizable property, gelatin methacryloyl (GelMA) was adopted for the design of the delivery system with chlorhexidine (CHX) to promote the controllable release.^47^ The infected root canal typically has high level of matrix metalloproteinase (MMP), which can degrade the GelMA to release the inner CHX in a responsive way. In addition, some GelMA‐based scaffolds have been acted as stem cells carriers to achieve pulpal revascularization.^48^ CS is a natural polymer with abandon positive charge, which can serve as an antibacterial hydrogel to kill the bacteria. Meanwhile, benefitting from the highly biocompatible porous structures, CS has the ability to regulate the release of bioactive agents inside. Caballero‐Flores et al. fabricated a CS‐based hydrogel system, which had microparticulate dentin and genipin as the stem cells delivery system.^49^ The hydrogel could realize more protein release, and promote adhesion, proliferation and differentiation of stem cells. To avoid the potential risks from the stem cell transplantation, some cell‐homing strategies have been explored. Dental pulp stem cells (DPSCs)‐derived exosomes have been used in oral regenerative medicine with proliferation, migration, odontogenesis, and neurogenesis stimulating properties.^50^, ^51^ Chen et al. developed a DPSCs‐exos‐laden scaffold to recruit the loaded stem cells from the root tip.^52^ The results demonstrated that the exos could induce stem cells to regenerate connective tissues, holding potential to treat endodontic defects associated with a wide range of endodontic diseases (Figure 3).

## 4 HYDROGELS APPLICATION IN THE APICAL PERIODONTITIS

**FIGURE 4:**
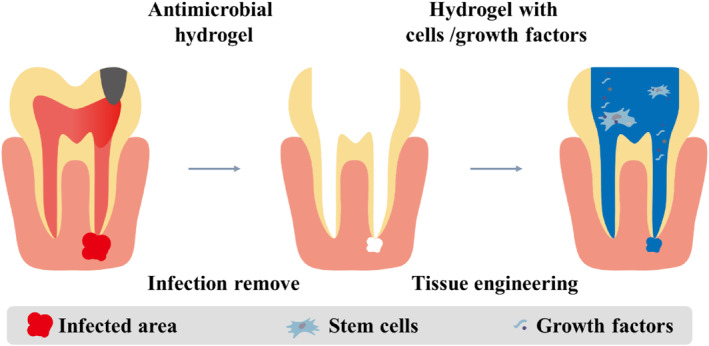
Hydrogel application in apical periodontitis. After removing the infection in the dental canal and apical region, the hydrogel‐based strategy can enhance the tissue regeneration in the local site.

With inappropriate treatments, the endodontics may develop into apical periodontitis, which is manifested by inflammatory damage breaching the apical foramen and affecting the periapical tissues. As an injectable scaffold, the hydrogel can deliver several ingredients to relieve local inflammation and promote bone repair (Figure 4). With the ability to remove infection, lysis necrotic tissue, and facilitate hard tissue formation, calcium hydroxide (Ca(OH)_2_) has been used in the treatment process of endodontists and periapical diseases for a long time. To reduce the potential cytotoxic effects on adjacent tissues from burst release of Ca(OH)_2_, Li et al. constructed microcapsules consisting of CS and Ca(OH)_2_ to deal with a mandible bone defect in rabbits.^53^ The resultant microcapsules showed pH‐responsive properties and enhanced antibacterial and osteogenic activities owing to the CS and their core–shell structure. Otherwise, some in vivo research proved that the addition of CS scaffolds in the regenerative strategy had no impact on the improvement of the form of new tissues.^54^ The addition of bioactive materials is regarded as a useful way. The DPSCs with accessibility have been explored in the tooth regenerative procedures.^55^ Many researches taken DPSCs with hydrogel scaffolds to act as mandible defect filling materials. The in vivo results demonstrated that the hydrogel could regenerate tooth pulp like tissue and contribute to complete root maturation with apical periodontitis.^56^

## 5 HYDROGELS APPLICATION IN THE PERIODONTITIS

**FIGURE 5:**
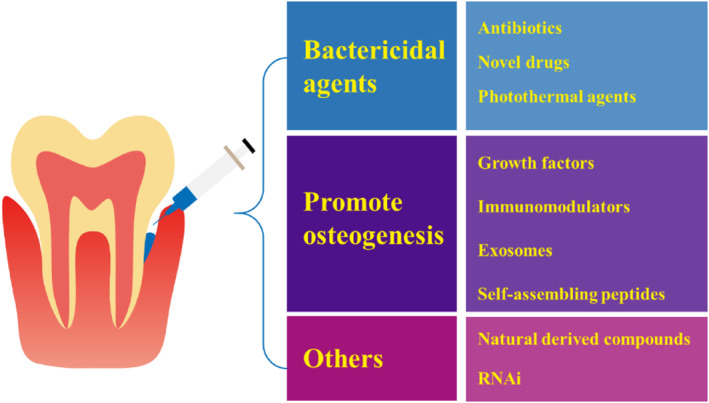
Hydrogel‐based bacteria removal and tissue repair application in periodontitis.

As a leading reason of the teeth loss, periodontitis brings much inconvenient to human in daily life. Bacterial infection plays the primary role in the essential periodontitis. Specifically, after the accumulation of pathogens and their production, the immunity‐relative cells can be active in the surrounding tissue, accompanied by tissue inflammation and gingivitis. Without suitable intervention, the immunoreaction will further result in hard and soft tissue defects, that is, periodontitis. When the periodontal bone is excessively defected, the teeth will loosen. According to the essential and development of the periodontitis, it is the key to removing bacteria and promoting local tissue repair in the treatment of periodontitis (Figure 5).

### 5.1 Bactericidal agents' delivery

#### 5.1.1 Conventional drugs

Some drugs have shown adjuvant effects in the treatment of periodontitis. Drugs loading with hydrogels can achieve the long‐time infiltration of the medication into the periodontal pockets and reduce the cycle of reapplication. Dong et al. developed an injectable hydrogel with CS, metronidazole (MTZ) and PVA, which could adhere to the tooth and sustainedly release the MTZ. The in vivo data showed that the topical delivery exhibited desirable antibacterial capacity towards experimental periodontitis.^57^ Wang et al. employed the thermo‐reversible polyisocyanopeptide (PIC) to deliver the doxycycline (DOX) and lipoxin A4 (LXA4). By combining the DOX's bactericidal effect and LXA4's inflammation regression, this PIC/DOX/LXA4 hydrogel decreased the subgingival bacterial number and interleukin‐8 (IL‐8) level in dogs.^58^ Zhao et al. fabricated hydrogel consisting oxidized dextran (OD) and phenylboronic acid‐functionalized poly (ethylene imine) (PBA‐PEI) as local drug delivery system (LDDS). The resulting injectable system allowed for simultaneous enhancement of doxycycline and metformin loading efficiency via B‐N coordination.^59^ This ROS‐triggered drug release strategy showed a remarkable antibacterial effect on *Staphylococcus aureus*, *Escherichia coli*, and *Porphyromonas gingivalis*. To gain the goal of the responsive release of the drugs, some environment responsive systems were developed.^60^ Guo et al. synthesized a responsive hydrogel with diacrylate‐containing PEG‐based bio‐scaffolds and a cysteine‐terminated peptide crosslinker, giving the resultant production with matrix metalloproteinase 8‐responsive release behavior.^61^

#### 5.1.2 Novel drugs

Several new drugs were developed to act as promising antibacterial agents. Guo et al. successfully synthesized an injectable versatile piezoelectric hydrogel (PiezoGEL) containing barium titanate (BTO) fillers for the nonsurgical management of periodontitis.^62^ This novel biomaterial has unique antimicrobial and bone tissue regeneration properties. With the use of a minimally invasive tip, the PiezoGEL can be locally injected into affected periodontal pockets and a graft can be formed in situ by light curing.

#### 5.1.3 Photothermal agents

As an effective noninvasive therapy, photothermal therapy has been widely applied in the tumor ablation and bacterial killing. By converting light energy to the thermal energy, the local hyperthermia can directly destroy the bacteria. Zhang et al. constructed a photoactive nano‐antibiotic platform, named PCM@GNC‐PND, by incorporating a gold nanocages (GNC) and phase‐change materials (PCM) and thermosensitive polymer poly(N‐isopropylacrylamide‐co‐diethylaminoethyl methacrylate) (PND).^63^ The GNC exhibited outstanding photothermal conversion effects due to its intense absorption in the near‐infrared (NIR) region, and its hollow inner structure made it a beneficial carrier for various antibiotics like tetracycline. The on‐demand drug‐release hydrogel system precisely realized the synergistic effect of photothermal and antimicrobial drugs. Unlike the metal‐based photothermal agents, polydopamine (PDA) is a kind of material with appealing photothermal conversion efficiency and is more economical, biofriendly and accessible.^64^ Tong et al. reported a curdlan/PDA composite hydrogel system to achieve the photothermal therapy (PTT) antibacterial and routine controllable release of antimicrobial upon the NIR irradiation.^65^

### 5.2 Promote osteogenesis

Periodontal tissue regeneration is the final goal of the treatment of the periodontitis, while the bone repair is the foundation. To deal with bone loss in the periodontitis, a series of hydrogels with different functions were developed.

#### 5.2.1 Growth factors

Delivering growth factors is the most direct way to promote bone regeneration. An injectable thermosensitive hydrogel was prepared using CS, disodium glycerophosphate (b‐GP) and gelatin, which sustained the release of aspirin and erythropoietin (EPO), which exerted anti‐inflammatory and tissue regenerative pharmacological effects.^66^ The aspirin released in the early stage could facilitate the regeneration function of EPO in the following step. In another study, researchers synthesized a smart gingival peptide‐responsive hydrogel (PEGPD@SDF‐1) as an environmentally responsive carrier for the release of drugs on demand.^67^ Several studies have shown that the SDF‐1 (stromal cell derived factor‐1) could recruit the periodontal ligament (PDL) stem cells to realize the bone defect repair.^68^ The designed hydrogel with gingipain‐responsive property could control inflammation and realize in situ bone regeneration.

#### 5.2.2 Immunomodulators

Periodontitis can be recognized as the result of the immunity response between the host and the pathogens. Therefore, the immunomodulators may make sense in treatment. The accumulation of the macrophages is critical in the periodontitis progression, which can secret cytokines and proteases to accelerate the periodontal tissue degeneration and damage. Many agents like interleukin‐4 and metformin have been proved to activate M2 macrophage polarization to facilitate the tissue repair. Zhang et al. developed a modular GelMA patch that delivered both antibiotics and cytokines (IL‐4 and TGF‐β) into the periodontal tissue to gain the immunomodulation and tissue regeneration.^69^ The sustained release of cytokines induced the macrophage repolarization and Treg formation (regulatory T cells), providing a pro‐healing microenvironment for tissue regeneration. Gong et al. designed a metformin‐loaded patch consisting of silk fibroin/gelatin showing immunomodulatory effect.^70^ The strategy accelerated the periodontal bone and ligament regeneration in diabetic rats. Dendritic cells (DCs) are also critical regulators of T cells and play a role in the pathology. Studies showed that alginate hydrogel loaded with the thymic stromal lymphopoietin (TSLP) and granulocyte‐macrophage colony‐stimulating factor (GM‐CSF) could upregulate the DC and FOXP3+ cells' number, which would hold promise to treat the upregulated pathologic inflammation of periodontal disease.^71^

#### 5.2.3 Exosomes

As a new intercellular communication mode, exosomes can deliver DNA, proteins, miRNAs and other bioinformatic mediators, and perform biological functions such as antigen presentation, immunomodulation, and repair of damaged tissues.^72^ To prepare an exosome carrier, Shi et al. took laponite and gelatin as a hydrogel system to form an injectable biological scaffold.^73^ Extracellular vesicles derived from dental follicular cells facilitate the maintenance of alveolar bone levels in both early and late stages of periodontitis in animals, paving a new way to cell‐based therapy for periodontal tissue engineering. To modulate the macrophage phenotype, Shen et al. developed a DPSC‐exo‐incorporated CS hydrogel (DPSC‐Exo/CS) to alleviate periodontitis in mice.^74^ The results demonstrated that the therapy could speed the alveolar bone and periodontal epithelium healing. And the further analysis showed that the local tissues were ameliorated by inhibiting periodontal inflammation and regulating the immune response.

#### 5.2.4 Self‐assembling peptides

SAPs have drawn a growing interest owing to their unique properties in the field of regenerative medicine. The SAPs can form three‐dimensional hydrogels like natural biological extracellular matrices to provide support for cell attachment proliferation and differentiation. Besides, the SAPs have shown great application potential in the biomineralization and wound healing. P11‐4 is a biomimetic material with the property of ECMs of the mineralized tissue and has been used in the early caries treatment. To study the application of P11‐4 hydrogel on PDL and alveolar bone regeneration, El‐Sayed et al. applied the hydrogel on the rats with periodontal defects.^75^ The results demonstrated that the periodontal fibrous tissue was better organized in the treatment group, and a marked degree of increase in the length of the functional periodontal ligament was observed histomorphometrically, suggesting that SAP P11‐4 is a promising drug candidate for periodontal regenerative therapies.

### 5.3 Other

#### 5.3.1 Natural derived compounds

Many active compounds extracted from Chinese herbal medicine have the antioxidative and anti‐inflammation properties, which can be used in the treatment of the periodontitis. Naringin is an active compound that can inhibit inflammation caused by bacteria and lipopolysaccharides (LPS), and is therefore considered a natural alternative for the treatment of periodontitis. To evaluate the efficacy of naringenin in the treatment of periodontitis in vivo, Chang et al. added naringenin to a CS‐based hydrogel with thermosensitive and pH‐responsive properties.^76^ The naringin‐loaded hydrogel could reduce bond loss and inflammatory infiltration, inhibiting the induction of experimental periodontitis.

#### 5.3.2 RNAi

Bone damage is the most severe result of dental diseases, eventually resulting in tooth loss, during which the osteoclast usually exhibits abnormal activity. The receptor activator of NF‐κB (RANK) is a key point in the osteoclastic process. It is reasonable to inhibit the osteoclast via RANK inhibition to prevent local bone loss. To keep siRNA from degradation, Ma et al. developed a RANK siRNA‐loaded CS hydrogel, aiming to establish an RNAi delivery system to deal with periodontitis.^77^ The hydrogel dramatically enhanced the fluorescent signal from one day to 14 days, providing proper repositories and carriers for localized sustained delivery of siRNA for potential treatments.

## 6 HYDROGELS APPLICATION IN THE ORAL MUCOSAL DISEASES

**FIGURE 6:**
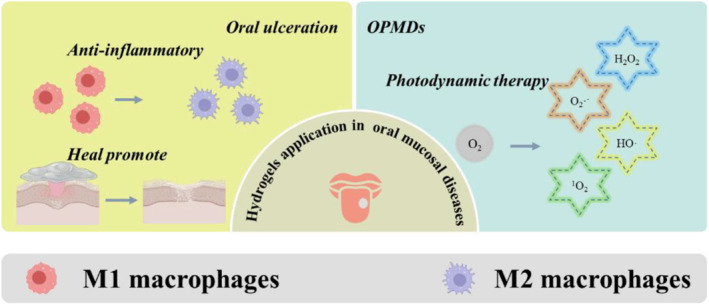
Hydrogel application in oral ulceration and oral potentially malignant disorders.

The oral mucosa is the mucous membranes arranged inside the mouth, acting as a barrier to the oral environment. The oral mucosa can protect the oral tissue from the physical, chemical and biological insults during the daily life. When diseases occur in the mucosa, the unpleasant feelings dramatically affect the quality of life. The oral ulceration and oral potentially malignant disorders (OPMDs) are the major lesions in the oral mucosa. Intraoral drug delivery in highly dynamic and humid environments presents many problems and challenges. Even worse, the movement from speech, swallowing and facial expression can also impact the adhesion of the topical agents. Among solutions, powders, ointments, polymer films and hydrogels, the hydrogels with enhanced adhesive ability bring new hope in the treatment of oral mucosal disease (Figure 6).

Oral ulceration is the most prevalent mucosa defect caused by trauma, infection and other uncertain reasons, affecting approximately 25% of young adults.^78^, ^79^ To address the ulceration, developing hydrogels with anti‐inflammatory and healing promoting effects is important. Meanwhile, the wet‐adhesive property as another essential parameter should be considered. Marine mussels with excellent underwater adhesion properties provide the ideal concept for oral application because the mouth contains a lot of saliva.^80^, ^81^ The adhesive properties of mussels are attributed to their rich content of the catechol amino acid 3,4‐dihydroxyphenylalanine (DOPA), an amino acid that supports adhesion.^82^, ^83^, ^84^ Inspired by this promising concept, An et al. fabricated a Janus gelatin‐PDA‐nanoclay (GPC) hydrogel.^85^ Through the synergistic physical and chemical effects of gelatin, nanoclay and dopamine, this hydrogel showed controlled adhesion and toughness, which improved the healing ability of oral ulcers and the long‐lasting therapeutic effect of dexamethasone. The phenolic moieties pyrogallol is another agent that can provide underwater adhesion. Inspired by this approach, Choi et al. conjugated pyrocatechol molecules with a pectin polymer (Pec‐PG) to make a highly adhesive pectin hydrogel for the treatment of oral diseases.^86^ The sprayable Pec‐PG illustrated good mucoadhesion properties. By loading the triamcinolone acetonide, the Pec‐PG could accelerate the healing process of oral ulcers in animal models. To avoid side effects from the hormonal drugs, Zhang et al. developed a polymerized ionic liquids (PILs)‐based diclofenac sodium‐loaded patch.^87^ The PILs display wide applications in the medical field, such as antibacterial agents, wound dressing materials, and drug carriers.^88^, ^89^, ^90^ By loading the diclofenac sodium, the patch demonstrated excellent biocompatibility, antimicrobial, disinfection, anti‐inflammatory and wound healing attributes in infected oral ulcers in vivo. Also, the strectchability and self‐healing attributes made the patch adapt to the complex oral environment. The light curing hydrogel shows a quick cure process, making it more convenient for oral mucosal repair. Liu et al. utilized decellularized human amniotic particles‐loaded GelMA as a tissue replacement to stimulate rabbit oral mucosal wound healing.^91^ Decellularized human amniotic cells have various growth factors that promote cell proliferation and differentiation and accelerate tissue healing. By loading the decellularized human amniotic particles, the hydrogel upregulated the angiogenesis and collagen expression in rabbit oral mucosa. Some hydrogels own microphage polarization property, promoting M2 cell polarization for anti‐inflammatory effect. Taking this advantage, Zhang et al. developed a photo‐crosslinking hydrogel adhesive for oral mucosa repair.^92^ The photoresponsive, cyclic o‐nitrobenzyl‐modifed hyaluronic acid hydrogel (HA‐CNB) could generate different active groups for nonradical crosslinking in less than 5s, forming a thin gel. The high‐molecular‐sized HA was taken as the base of the gel for anti‐inflammation. The prepared hydrogel could effectively protect the damaged area for one day, promoting oral mucosal wound healing in both rats and pigs.

To improve the drug permeability inside the hydrogel, microneedles as a new drug delivery strategy have been developed.^93^ The microneedles could penetrate the surface of the skin or mucous to deliver drugs directly to the local sites in a minimally invasive way.^94^ Based on the monolayer MNs, Meng et al. designed double‐layer dissolving microneedles with hyaluronic acid methacryloyl, HA, and polyvinylpyrrolidone to deliver two drugs for oral ulcer treatments.^95^ The microneedles could penetrate the surface of the oral mucosa, following the effectively release of the loaded drugs into mucosa tissue, achieving sequential drug delivery. This hydrogel microneedle‐based drug delivery system may be used as an efficient, needle‐free alternative injection way. To improve the adhesive ability, several microneedles with well‐designed structures were fabricated.^96^, ^97^, ^98^ For example, inspired by the octopus, Zhu et al. developed a wet‐bonding microneedle patch (silk fibroin‐pluronic‐F127‐poly(N‐isopropylacrylamide)) with a unique flexible cup structure for controllably delivering dexamethasone for oral ulcers.^99^ Such a structure endowed the microneedle patch with physical adhesion, allowing the drug‐loaded core to contact the ulcer.

OPMDs are premalignant lesions with a high risk of malignant transformation to the oral cavity cancer.^100^ Generally, 15 kinds of oral mucosal disorders are defined as OPMDs, ranging from oral leukoplakia (OLK), oral erythema (OE), oral submucosal necrosis, and proliferative verrucous leukoplakia (PVL).^101^ More evidences have demonstrated that the conventional therapies such as surgery section lead to unsatisfactory results, whereas the high recurrence rate and mucosal dysfunction hinder their application. Currently, the 5‐aminolevulinic acid (ALA)‐based photodynamic therapy acted as a promising option for patients with OPMDs and early‐stage oral squamous cell carcinoma, while the complex procedures and inadequate concentration offer new demand for the delivery system.^102^, ^103^ To improve the wet adhesion of the ALA‐loaded hydrogel, Wang et al. developed a poly(acrylic acid) (PAA)‐CS‐ALA‐based hydrogel patch.^104^ The prepared hydrogel formed a rapid temporary cross‐linking with wet surfaces of the oral mucosa, and the subsequent covalent cross‐linking further enhanced adhesion properties.^105^ The resultant hydrogel displayed high anticancer efference on OPMDs in vitro and in vivo upon 635 nm laser application with excellent comfort and acceptability.

## 7 HYDROGELS APPLICATION IN THE SALIVARY GLAND DISEASES

**FIGURE 7:**
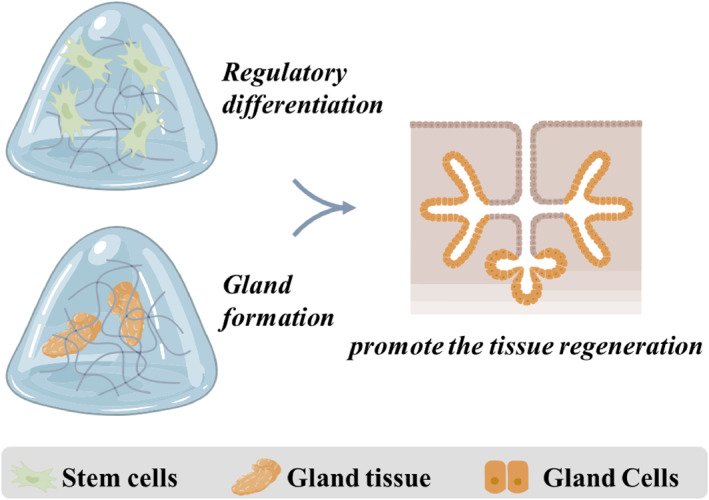
Hydrogels with stem cells or tissues enhance gland‐like tissue formation in treating salivary gland diseases. The factors encapsulated in the hydrogel can regulate stem cells differentiation and gland formation to promote the tissue regeneration.

Saliva plays a key role in maintaining the oral cavity environment, with functions such as food mastication, antibacterial effect and teeth re‐calcification. When impacted by the aging, autoimmune diseases, or radiotherapy damage, the salivary hyposecretion occurs. The xerostomia may also be accompanied by a high risk of dental caries, periodontitis, oral candidiasis, and dysgeusia. Some people can feel a burning sensation, which dramatically impacts their quality of life. Notably, the current clinic treatments are focused on relief symptoms, which are palliative but not reparative. The application of artificial saliva only targets temporary relief and owns huge inconvenience. Upregulated aquaporin‐1 with gene therapy can enhance the secret salivary flow in irradiated cells, but the vital constituents also lack, hindering its wide application in the clinic. With the development of the bioengineering, the fabrication of the salivary gland tissue is promising for gland regeneration, where the hydrogels are an ideal candidate to act as an extracellular matrix (Figure 7).

The epithelial cells from the submandibular gland can self‐organize to gland‐like tissue under suitable environments.^106^ Ogawa et al. have demonstrated a hydrogel coculture system from collagen gel with mesenchymal and epithelial cells to generate submandibular gland‐like tissue.^107^ The bioengineered submandibular gland could secrete saliva in response to stimulation in vivo. To expand the application of the mesenchymal stem cells (MSCs), several efforts have been made to clarify the influence of MSCs. Much research has revealed that the conditional medium derived from MSCs could enhance multi‐lumen formation in mouse submandibular clusters growing on fibrin hydrogel.^108^, ^109^, ^110^ During the morphogenesis of the salivary gland in 3D hydrogels, gland cells' migration and branching are crucial, which are the foundation of the functions of the glands. To control the action mode of the two morphogenetic signals, Samuel et al. developed a hydrogel system that could maintain the length and cellularity of branches as well as the formation of new nodes/clusters.^111^ The hydrogel from fibrin could deliver the Fibroblast Growth Factor‐7 (FGF‐7) and Fibroblast Growth Factor‐10 (FGF‐10) with different presentation and delivery modes. In addition, to avoid the side effects of the uncontrollable release of relative factors, the modified hydrogel with growth factor was fabricated.

To enhance the aggregation of the salivary gland tissue, KP24 (a peptide with repeating proline and lysine sequences) was added to the alginate through carbodiimide chemistry. Ikeda et al. proved that the KP24‐immobilized alginate significantly enhanced the SMG growth by increasing neuronal growth and enhancing neural innervation.^112^ Another alginate‐based novel hydrogel system was modified with egg white, providing extracellular matrix (ECM)‐like proteins to offer necessary nutrition. Zhang et al. illustrated that the egg white‐alginate porosity, strength and stiffness could be tuned with the different concentrations, which may be used as a potential source for tissue engineering of salivary spheroid and cell transplantation.^113^ To reduce the side effect induced by the exogenous, autologous‐derived hydrogel may provide a suitable choice. El‐Latif et al. injected the autologous fibrin hydrogel loading with allogenic gingival margin‐derived stem cells (GMSCs) to evaluate the regenerative effect on submandibular salivary glands.^114^ The results showed that the regeneration of ductal, acinar, and myoepithelial cells was better in the relative groups, and the proliferating cell nuclear antigen and *α*‐smooth muscle actin were also upregulated in the partially dissected submandibular salivary glands.

In conclusion, the hydrogel can promote the tissue regeneration through delivering the factors and stem cells, providing an opportunity in the treatment of salivary gland diseases.

## 8 HYDROGELS APPLICATION IN THE ORAL TUMOR

### 8.1 Chemotherapy

Chemotherapy, as a traditional method to conquer tumor, has been applied for several years. The used chemotherapy drugs showed low tumor targeting and side effects, demanding effective local drug delivery strategies to enhance the results. Doxorubicin (Dox), cisplatin (CDDP) and paclitaxel are well‐known drugs used in the clinic. Loading these drugs into hydrogels was demonstrated to be effective for dealing with the oral tumor. To fabricate a controlled drug delivery system, Li et al. fabricated a hydrogel with PEG and polycaprolactone (PCL) to deliver histone deacetylase inhibitors (SAHA) and CDDP.^115^ By direct intratumoral injection, the hydrogel could induce an antitumor activity against xenografts in a murine model, providing a useful strategy to treat oral cancer and other solid tumors. Local injection of hydrogel with Dox and celecoxib (Cel) obtained significant efficacy in tumor growth inhibition.^116^ Besides, there are hydrogels that have inherent antitumor effects without adding more drugs. Zhao et al. developed isoguanosine‐borate‐guanosine (isoGBG) supramolecular hydrogels for cancer therapy.^117^ The prepared hydrogel with distinctive self‐healing, injectable and degradable capabilities exhibited superior anti‐oral cancer and prevented the post‐operative recurrence, making it an ideal candidate for future clinical treatment of cancer. In addition to the injectable hydrogel, film‐like hydrogel also has been widely used as oral cancer treatment patches.^118^

### 8.2 Phototherapy

PTT has been widely investigated for cancer treatment. Considering the superficial tumor in the oral region, PTT has gained much attention to treat oral tumors. By converting the light energy to the heat, the photothermal agents (PTAs) induced consequent hyperpyrexia can directly destroy the tumor cells with minimal lesion. Many PTAs, including organic agents and inorganic nanoparticles (NPs) have been developed to conquer the tumor with/without surgery. The poor biocompatibility and non‐targeted accumulating of PTAs, which may have unforeseen adverse side effects on normal tissues and organs, offering new demands on the delivery strategy. Considering the injectable property of the CS hydrogel, Su et al. constructed Ag_3_AuS_2_ NPs loaded hydrogel network to achieve effective therapy toward tongue cancer treatment with good biocompatibility and ultra‐strong photothermal effect.^119^ This injectable hydrogel could be precisely applied around the tongue tumor site via peritumoral delivery. Also, the hydrogel can remain in situ for a long time, enhancing the local photothermal effect from the integrated PTAs. The well‐designed strategy offered no negative side effects on surrounding tissues and inhibited potential local tumor recurrence, holding potential for clinical oral cancer therapy. In addition to the single therapy, the PTT can be used together with chemotherapy. Wu et al. developed an injectable and NIR‐responsive hybrid system for chemophotothermal therapy.^120^ The hydrogel was taken light‐responsive mesoporous silica nanoparticles (MSNs) as Dox vehicles, which were injected into an IR820/methylcellulose hydrogel network. The NIR radiation could destroy tumor cells with induced photothermal effects and achieve controlled Dox release, gaining the chemotherapy.

Some photosensitizers can produce much cytotoxic singlet oxygen and free radicals, inhibiting proliferation and inducing apoptosis of tumor cells, called PDTs. Topical PDT mediated by ALA has been applied in the clinic for oral potentially malignant disorders treatment and oral tumors. Despite this progress, some shortcomings remain, including conveying efficiency, inferior comfort, and susceptibility to salivation. To address these issues, Wang et al. designed a highly adhesion‐strength network hydrogel patch consisting of PAA‐CS‐ALA.^104^ The hydrogel patch could fast and stably adhere to the wet oral mucosa and convey ALA, effectively ameliorating OPMDs in vitro and a hamster oral carcinogenesis model. In addition, recruited volunteers also proved the practicability and comfort of the PACA patch in vivo. The hydrogel‐based PDT could be a user‐friendly treatment for OPMDs and oral tumor.

### 8.3 Immunotherapy

**FIGURE 8:**
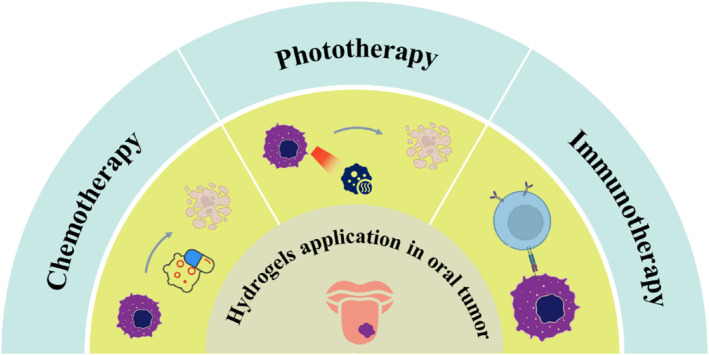
Hydrogel therapy in the treatment of oral tumors includes chemotherapy, phototherapy, and immunotherapy. During the chemotherapy process, the chemotherapeutics can kill the tumor cells with the growth and division interfering. With the involvement of photosensitizers, light can be converted into heat or produce reactive oxygen species to kill tumor cells. Immunotherapy can kill tumor cells by enhancing the function of immune cells.

Immunotherapy has shown considerable therapeutic effects in the oral tumors. However, the response rate of OSCC to pembrolizumab immunotherapy was just 14.6%, which may be due to the suppressive immune microenvironment. Several recent studies have documented an affiliation between the modulation of commensal bacteria and the responsiveness of the host immune system.^121^, ^122^ To improve the effectiveness of cancer therapies, Zheng et al. designed a silver NP (AgNP)‐based hydrogel, enhancing tumor immunotherapy via the modulation of microbiota in the oral cavity.^123^ By increasing the number of endogenous and exogenous beneficial flora, the strategy could synergize with checkpoint inhibition via programmed death‐1. The research suggested that biomaterials could be engineered to modulate the human microbiota, thereby enhancing anti‐tumor immune responses (Figure 8).

## 9 HYDROGEL APPLICATION IN OTHER ORAL‐RELATED FIELDS

Apart from the application mentioned above, there are also some related applications in the oral and maxillofacial region. Bone defects widely appear from trauma, tumor, and infections, bringing much inconvenience to the patients. When bone loss occurs in the jawbone, it is often accompanied by tooth loss and other physical disabilities. Sufficient bone repair is a crucial point for successful treatment. Taking inspiration from the native craniofacial bone, Shi et al. developed a GelMA‐based hydrogel with BMP‐2 and calcium phosphate oligomer to improve the repair process.^124^ By a well‐designed structure, the neo‐bone formation was accelerated with gradient densities. The hierarchical graft provides a new treatment for craniofacial bone regeneration. The jawbone cannot function without the muscles, and nerve damage in the maxillofacial area can affect muscle movement, severely affecting the face and quality of life. The most common cause of facial muscle injury is due to facial nerve damage from trauma or tumors. Many hydrogel‐based therapies have been explored to enhance peripheral nerve defect repair. By loading the growth factors, the fabricated hydrogel could increase the number of blood vessels and promote functional recovery.^125^ Also, several bioprinting methods and bioinspired hydrogel actuators have shown great application potential in the regeneration of muscle tissues.^126^, ^127^ Scarless healing of the wounds is essential in facial skin. Hydrogel‐based therapies also show great promise in this regard. The rational drug delivery and mechanical design of the hydrogel can lead to accelerated healing and reduced collagen deposition.^128^, ^129^

All this research broadened the application scenarios of hydrogels, and we believe that hydrogel‐based treatments become more translational and produce clinically relevant, gratifying results. But, compared to the hydrogel applied to other parts of the body, hydrogel used in oral disease have to address some special situations summarized as follows: (a) The frequent movements of the mouth (chewing, speaking, and swallowing) cause frequent deformation of the hydrogel, which places high demands on the adhesion of the hydrogel. (b) Owing to the unique structure of the oral cavity, good biocompatibility of the hydrogel is required. Poor biocompatibility can lead to mucosal immune reactions, and harmful functional monomers can enter the body through the digestive tract. (c) The oral cavity has a complex microbial environment, variable temperature, and abundant enzymes, which require hydrogels to have good stability.

## 10 OUTLOOK

The past decades have witnessed the advancements of functional hydrogel application in several diseases. In this review, we have summarized the latest progress of functional hydrogel‐based therapy in treating oral disease. Especially, to meet the requirements of oral disease, hydrogels are endowed with advanced functions such as antibacterial activity, promote osteogenesis, immunomodulation, and tissue engineering. These functions have significantly improved their performance and made them exhibit promising prospects in dealing with the disease in the oral cavity. Despite the great progress, several problems confusing the treatment of oral diseases still remain and need to be addressed.

Advances in materials and therapeutic concepts have resulted in tooth implants being used more and more widely in clinical practice. Although implant restorations are able to bring better esthetic effects and functionality compared with conventional restorations, they are inevitably accompanied by the emergence of periimplantitis. Unlike periodontitis, periimplantitis has no biological activity and is more difficult to treat. Taking inspiration from hydrogels for the treatment of periodontal diseases, hydrogels, especially injection ones, would be potential for dealing with periimplantitis by delivering agents to clear inflammation and promote tissue regeneration.

Furthermore, diagnosing hard tissue infections in the oral cavity is currently more dependent on doctor's examination and X‐ray tests. On this side, it puts high demands on doctors, and on the other side, the frequent X‐ray test will bring potential health hazards. The construction of diagnosis and treatment integration hydrogels can be a good solution to this problem. In this way, patients can understand their oral health condition by themselves and can also find out the health problems as early as possible.

The third issue concerns the performance optimization of hydrogel materials. Specifically, in order to meet the particular physiological environment of the oral cavity, the prepared hydrogels should satisfy the requirements of good anti‐swelling properties. The current preparation methods, whether ionic cross‐linking or photocrosslinking hydrogels, inevitably have swelling properties due to the presence of scaffold networks. Through the addition of particular metal ions or the formation of a double network or even multi‐network structure, the swelling of the hydrogel can be significantly reduced.

In conclusion, we have focused on the advanced functions and application of functionalized hydrogels in the treatment of oral diseases. Ranging from the anti‐bacterial, anti‐inflammation, remineralization, and tissue engineering, the functional hydrogels have been widely applied to deal with oral diseases. With the shift in diagnostic concepts and treatment modalities, new concerns are placing new demands on hydrogel therapy. Despite the challenges, advances in materials science, manufacturing technology, and clinical medicine will drive the development of the next generation of functionalized hydrogels. We believe that future functionalized hydrogels will bring new opportunities for treating and diagnosing oral diseases.

## AUTHOR CONTRIBUTIONS

Yu Wang provided the idea; Chuanhui Song wrote the manuscript; Rui Liu, Yile Fang, and Hongcheng Gu revised the manuscript.

## CONFLICT OF INTEREST STATEMENT

The authors declare that there are no competing interests.
